# Somatic Mutations of *lats2* Cause Peripheral Nerve Sheath Tumors in Zebrafish

**DOI:** 10.3390/cells8090972

**Published:** 2019-08-25

**Authors:** Zachary J. Brandt, Paula N. North, Brian A. Link

**Affiliations:** 1 Department of Cell Biology, Neurobiology and Anatomy, Medical College of Wisconsin, Milwaukee, WI 53226, USA; 2 Department of Pediatrics, Medical College of Wisconsin, Milwaukee, WI 53226, USA

**Keywords:** Lats2, Hippo, Yap, Taz, peripheral nerve sheath tumor, schwannoma, zebrafish

## Abstract

The cellular signaling pathways underlying peripheral nerve sheath tumor (PNST) formation are poorly understood. Hippo signaling has been recently implicated in the biology of various cancers, and is thought to function downstream of mutations in the known PNST driver, *NF2*. Utilizing CRISPR-Cas9 gene editing, we targeted the canonical Hippo signaling kinase Lats2. We show that, while germline deletion leads to early lethality, targeted somatic mutations of zebrafish *lats2* leads to peripheral nerve sheath tumor formation. These peripheral nerve sheath tumors exhibit high levels of Hippo effectors Yap and Taz, suggesting that dysregulation of these transcriptional co-factors drives PNST formation in this model. These data indicate that somatic *lats2* deletion in zebrafish can serve as a powerful experimental platform to probe the mechanisms of PNST formation and progression.

## 1. Introduction

Peripheral nerve sheath tumors (PNSTs) are relatively uncommon, making up just 8.6% of all primary brain or central nervous system tumors reported in 2011–2015 [1]. Based on these numbers, it was estimated that an additional 7500 new cases would be reported in 2018, representing one in every 50,000 people [1]. PNSTs are comprised of multiple tumor classifications including schwannoma, neurofibroma, perineurioma, and malignant peripheral nerve sheath tumors (MPNSTs) [2,3]. Nearly all of these tumors are benign (99.2%); however, they can result in loss of nerve function and/or neuropathic pain [4]. Although rare, the malignant subset of these tumors, termed MPNSTs, have poor prognosis for patient survival [5,6].

Very few genetic alterations are well recognized as drivers of PNST formation, and our understanding of these alterations and their underlying molecular mechanisms is far from complete. It is generally accepted that schwannomas, neurofibromas, and MPNTSs are derived from Schwann cells [7]. The subset of PNSTs with known genetic causes is termed neurofibromatosis. Neurofibromatosis commonly refers to three distinct and genetically determined dominant disorders, all leading to PNST formation with distinct characteristics [8]. These three disorders are referred to as neurofibromatosis 1 (NF1), neurofibromatosis 2 (NF2), and schwannomatosis. The aberrant genes that drive these disorders are *NF1*, *NF2*, and *INI1/SMARCB1*, respectively [8]. While the specific genetic variants causing these disorders are well defined, the molecular mechanisms by which they drive tumorigenesis remain incompletely understood and represent a key area of research. While approximately 90% of NF1 cases are sporadic, both syndromic and sporadic tumors can result from loss-of-function mutations in *NF1* [9]. *NF1* encodes neurofibromin 1, and acts as a Ras GTPase-activating protein, suggesting that improper regulation of Ras-related growth and proliferation may be an underlying cause in NF1 cases [10]. The *NF2* gene encodes for the tumor suppressor Merlin [11,12]. While the exact function of Merlin is not well understood, it has been shown to function as an upstream regulator of the Hippo signaling pathway, with direct implications in Schwann cells [13,14]. Finally, in schwannomatosis, the mutated gene of interest, *INI1/SMARCB1*, encodes a subunit of the SWI/SNF protein complexes that is known to function in chromatin remodeling [8]. In all three of these cases, the phenotypic variability is common and a high mutational load is noted [8]. This suggests that genetic modifiers as well as cooperating mutations exist and can alter the phenotypic outcomes of PNSTs. In support of this, while approximately half of MPNST cases are associated with *NF1* mutations, only 10% of NF1 cases eventually develop MPNST [15]. The other half of MPNST cases are sporadic, highlighting the importance of identifying other potential drivers of tumorigenesis.

As a signaling pathway downstream of Merlin/NF2, Hippo-Yap/Taz signaling is an obvious candidate for further study in PNST biology, and a logical pathway for targeted therapeutics. The Hippo pathway is primarily defined as a phosphorylation cascade composed of kinases Serine/Threonine Kinase 3 and 4 (Stk3 in zebrafish, Mst1/2 in mammals), and Large Tumor Suppressor Kinases 1 and 2 (Lats1/2), along with their respective adaptor/scaffolding proteins Salvador Family WW Domain Containing Protein 1 (Sav1 in zebrafish, WW45 in mammals), and MOB kinase activators 1 and 2 (Mob1a/b). After phosphorylation by Stk3/4, Lats1/2 regulates the main effectors of Hippo signaling transcriptional co-activators Yes-associated protein 1 (Yap) and its paralog, WW domain containing transcription regulator 1 (WWTR1/Taz), via phosphorylation. This phosphorylation leads to cytoplasmic sequestration of Yap/Taz through 14-3-3 protein binding, as well as subsequent ubiquitination and proteasomal degradation. Non-phosphorylated Yap/Taz translocate to the nucleus and, as they cannot bind DNA directly, associate with several families of transcription factors, most commonly TEA-Domain (TEAD) transcription factors, and both activate and repress expression of various genes.

Several studies have demonstrated roles for Hippo signaling in normal Schwann cell biology. Conditional knockout of *Nf2* in Schwann cells of mice led to hypomyelination and an increased Schwann cell number. This conditional knockout also affected injury response, as impaired axonal regeneration and remyelination were observed following sciatic nerve crush. Extending these findings to Yap signaling, the authors found that either mono- or bi-allelic deletion of *Yap* was able to rescue the impaired axonal regeneration in *Nf2* conditional knockouts [14]. Yap and Taz have since been identified as crucial to proper Schwann cell development. Several groups have shown that Yap and Taz can function redundantly in Schwann cells and are necessary for proper proliferation and myelination [16,17,18].

In recent years, Hippo signaling has also been shown to play a key role in aberrant Schwann cell biology, including PNST formation. Some of the first evidence of Hippo pathway involvement in PNSTs came through study of NF2. While working to uncover the molecular mechanisms of *NF2* mutant tumorigenesis, Li et. al. found that E3 ubiquitin ligase, CRL4^DCAF1^ promotes tumorigenesis of *NF2*-mutant cells by inactivating Lats1/2 and, hence, activating Yap [19]. Further study of Hippo signaling in the context of *NF2* mutation revealed that genetic and pharmacological inhibition of Yap led to decreased tumor cell proliferation and survival in *NF2* mutant cells and was able to reduce schwannoma tumor growth in mouse transplant models [20]. At the same time, several discovery-based studies of nerve sheath tumors identified alterations in Hippo pathway signaling. A proteomic screen for factors affected within sporadic human schwannomas revealed the activation of several receptors, including PDGFRβ, Her3, and Her2. Immunostaining and in vitro work in human schwannoma cell lines went on to suggest that expression of these receptors was under the control of Yap, and that proliferation in these tumors was linked to Yap signaling [21]. Furthermore, whole-exome sequencing of inherited and sporadic schwannoma reported *LATS1* mutations, suggesting that Hippo signaling could play a role in both types of schwannoma [22]. A more comprehensive and targeted study of sporadic schwannomas provided compelling evidence for the role of Hippo-Yap/Taz signaling in these tumors. Targeted sequencing of *LATS1* and *LATS2* in sporadic schwannoma revealed mutations in 1% and 2% of cases, respectively, suggesting that their mutation may be rare. However, promoter methylation of *LATS1* and *LATS2* was seen in 17% and 30% of cases, respectively. Overall, 76% of cases had at least one alteration in the *NF2, LATS1*, or *LATS2* gene. Of those cases, 43% of the tumors contained nuclear Yap expression by immunohistochemistry [23]. Transcriptomic data of vestibular schwannoma tumors from those patients also revealed deregulation of Hippo signaling [24]. These alterations may not be confined to schwannomas, as a case report of whole-exome sequencing in a single NF1 patient suggested that mutations in Hippo pathway-associated genes were overrepresented [25]. Strong evidence of Yap activation has also been seen in low-grade meningiomas and embryonal rhabdomyosarcoma [26,27]. *LATS2* mutations were also found in 11% of malignant pleural mesotheliomas and, of particular interest, co-occurring mutations of *NF2* and *LATS2* correlated with poor prognosis [28]. Consistently, these studies have found evidence for Lats1/2 activity in PNST formation, suggesting an important function in nerve sheath tumorigenesis. Cementing this role, combined conditional deletion of *Lats1* and *Lats2* within the Schwann cell lineage led to nerve-associated tumors in 100% of mice, and the development of a mouse model for MPNST [29]. The development of malignant tumors in this model also suggests that Lats1/2 and Yap/Taz signaling may be involved in malignant transformation of PNSTs. Consistent with this thought, a Yap/Taz conserved gene signature was more highly elevated in human MPNST samples than in NF1 or normal nerve samples [29]. Overall, these studies highlight a clear role for Hippo-Yap/Taz signaling in PNSTs. 

Zebrafish have emerged as a reliable and advantageous model organism for the study of various cancers [30,31]. This includes several models of MPNST [32]. An MPNST model in *nf1*- and *p53*-deficient zebrafish successfully demonstrates several advantages of zebrafish in this context. The lack of a fully developed immune system in early zebrafish larvae facilitated the transplantation of tumor cells without host rejection, while the large reproductive capacity of the fish allowed the investigators to test the effect of various drugs on tumor growth [33]. As many tumor suppressor genes play essential roles in developmental differentiation and proliferation, the germline mutation of these genes can lead to embryonic or larval lethality and difficulty in studying their role in cancer biology. Somatic inactivation via transcription activator-like effector nucleases (TALEN) and clustered regularly interspaced short palindromic repeats and caspase9 (CRISPR-Cas9) has proved valuable for the development of retinoblastoma and medulloblastoma models in zebrafish [34,35,36]. These advantages suggest that the development of a zebrafish model of Hippo pathway-associated PNST would be beneficial.

In this study, we provide evidence supporting the role for Hippo-Yap/Taz signaling in PNST formation by presenting a novel model of PNSTs in zebrafish. Somatic inactivation of the Hippo kinase Lats2 by mosaic CRISPR-Cas9 gene editing resulted in obvious tumorigenesis. Histological examination of the resulting tumors identified them as resembling PNST in morphology. This diagnosis was confirmed by immunostaining of known markers Sox10 and S100. Immunohistochemical analysis also revealed the tumors to be highly proliferative and to contain high levels of Hippo pathway effectors Yap and Taz. 

## 2. Materials and Methods

### 2.1. CRISPR Design

Clustered regularly interspaced short palindromic repeats (CRISPR) guides were designed against regions of zebrafish *lats2* exon5 and exon6 and *lats1* exon4 and exon7 using ZiFiT Targeter Version 4.2 (http://zifit.partners.org/ZiFiT, in the public domain). The targeted sequences were, 5′-GGAGCTAGTTATGGGGCTGA-3′ for *lats2* exon5, 5′-GGCATTGGGGCCTTTGGTG-3′ for *lats2* exon6, 5′-CCTCCCTATTCCATGCACC-3′ for *lats1* exon4, and 5′-GGGACTCTCGGGCGACGCAC-3′ for *lats1* exon7. CRISPR gRNA templates were generated by cloning annealed oligonucleotides with appropriate overhangs into *Bsa*I-digested pDR274 plasmid. CRISPR gRNAs were synthesized using a MEGAshortscript T7 Transcription Kit and purified using a mirVana miRNA Isolation Kit (Ambion, Austin, TX, USA). Zebrafish codon-optimized *cas9* was synthesized using a mMESSAGE mMACHINE Kit (Ambion) and polyadenylated using a Poly(A) Tailing Kit (Ambion). CRISPR gRNAs and *cas9* mRNA were co-injected into 1- to 4-cell zebrafish embryos from wild type ZDR fish maintained internally in the Link lab, at 12.5 ng/μL and 300 ng/μL, respectively, and surviving embryos were raised to adulthood before outcrossing to identify the founder fish carrying germline edits in *lats2*. Offspring from these fish were raised to adulthood, then fin-clipped for genotyping (see below for details). The resulting 1710-bp deletion mutant described here was identified via sequencing (Retrogen, San Diego, California, USA). This mutant allele is designated *lats2*^mw87^ (c.1048-2605del), where 1048–2605 denotes the deleted nucleotides of transcript: lats2-204 ENSDART00000139620.3 from GRCz11 genomic build.

### 2.2. Genotyping

**Figure 1:**
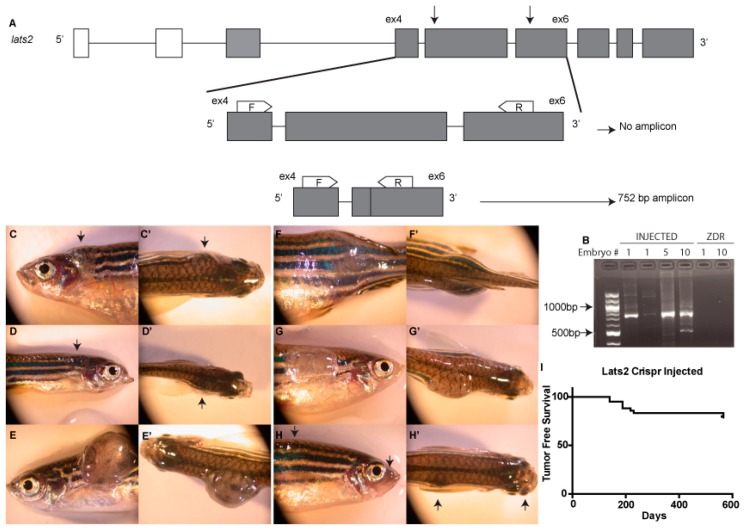
Injection of two *lats2* gRNAs leads to deletions and tumorigenesis. (**A**) Schematic depicting the zebrafish *lats2* gene and the PCR-based genotyping assay used to identify and differentiate between successful large deletion alleles and WT alleles. Arrows indicate the target sites of the gRNAs used. F refers to a forward primer; R refers to a reverse primer. (**B**) Agarose gel separation of PCR amplicons generated based on the genotyping assay depicted in (**A**). Amplicons of the correct size, indicative of a large genomic deletion, are present in *lats2* CRISPR-injected embryos, but absent in uninjected negative controls. Numbers above lanes represent the number of embryos used as the template DNA for each PCR sample. First lane contains a 100-bp ladder (NEB). (**C**–**H’**) Six examples of *lats2* CRISPR-injected fish that developed tumors by 6 mpf. Arrows denote tumors when not obviously visible. (**I**) Kaplan‒Meier plot of tumor-free survival in *lats2* CRISPR-injected fish.

Genomic DNA was extracted from zebrafish tissue using a Puregene Core Kit (Qiagen, Hilden, Germany). The genomic region containing the *lats2*^mw87^ mutation was amplified by PCR. The PCR protocol utilized primers flanking the expected deletion. The thermocycle conditions for detecting presence of a large deletion allele were designed using extension times allowing amplification of an amplicon ~750 bp in size, the expected size of an allele containing our targeted large deletion, but not for the WT allele amplicon of 2455 bp (Figure 1A). When detecting the presence of a WT allele, we either increased the extension time and screened for the presence of a 2455-bp amplicon, as well as the 750-bp amplicon, or included a third internal primer located within the sequence deleted in the *lats2*^mw87^ allele. A list of primers used for genotyping is provided below.

*lats2* Exon4 Forward Primer: 5′-CCTGAAACAGACTGGTAGC-3′

*lats2* Exon6 Reverse Primer: 5′-TTGAGTTGTGAGTCCATCGG-3′

*lats2* Exon5 Large Deletion Internal Primer: 5′-CATGTTTGTGGAGTAAGCAC-3′

*lats1* Exon4 Forward Primer: 5′-CAAGCGCTATTCTGGGAACT-3′

*lats1* Exon7 Reverse Primer: 5′-AAACTGAGCCAAGTCCTCCT-3′

### 2.3. Paraffin Histology

Adult fish used for paraffin histology were fixed in 10% neutral buffer formalin overnight at 4 °C. Large adult fish were cut open along their belly on the anterior to posterior axis to allow for better penetration of the fixative. Samples were then processed in paraffin on a Sakura VIP5 automated tissue processor (Sakura Finetek Europe, Flemingweg, The Netherlands) for histology and immunohistochemistry. After paraffin embedding, samples were sectioned at 4 μm (Microm HM355S, ThermoFisher Scientific, Waltham, Massachutesetts, USA) onto poly-l-lysine coated slides and air-dried at 45 °C overnight for any subsequent immunohistochemistry or routine H&E staining. Brightfield light microscopy images were taken using a NanoZoomer 2.0-HT (Hamamatsu Photonics K.K., Hamamatsu City, Shizuoka, Japan).

### 2.4. Immunohistochemistry

An optimal immunostaining protocol was developed with the use of a Leica-Bond Max Immunostaining platform. All slides were dewaxed prior to their optimal antigen retrieval protocol. All antibodies used a citrate buffer epitope retrieval (Leica Epitope Retrieval Solution 1, AR 9661). PCNA (sc56, 1:6000, Santa Cruz Biotechnology, Dallas, TX, USA), Yap1 (ab81183, 1:100, Abcam, Cambridge, UK), Sox10 (GTX128374, 1:100, Genetex, Irvine, CA, USA), Yap/Taz (D24E4, 8418, 1:200, Cell Signaling Technology, Danvers, MA, USA), and S100 (PA0900, ready to use, Leica, Wetzlar, Germany) were detected and visualized using Bond Polymer Refine Detection System (DS9800, Leica) with the addition of a DAB Enhancer (AR9432, Leica), with a Modified F protocol (primary antibody incubation: 15 min at room temperature). All slides were counter-stained with hematoxylin and cover-slipped using a synthetic mounting media. Omission of the primary antibody served as the negative control. 

### 2.5. Nomenclature

HUGO gene nomenclature for gene names and symbols was used within the text.

## 3. Results and Discussion

We targeted zebrafish *lats2* for deletion with CRISPR-Cas9 genetic editing techniques. Utilizing simultaneous injection of two CRISPR guide RNAs (gRNAs) targeting exons 5 and 6 of zebrafish *lats2*, we generated large deletions early in the coding sequence. These deletions remove the coding sequence that includes the serine threonine kinase domain, which is critical for the Lats2 protein function in canonical Hippo pathway phosphorylation. Providing evidence for large deletions of human *LATS2* in a clinical setting, the engineered mutations in zebrafish are similar to the large deletions reported in patients with malignant pleural mesotheliomas [28]. To determine whether we are able to create large deletions in zebrafish with this strategy, we designed a genotyping protocol utilizing primers flanking the expected deletion. The thermocycle conditions for our PCR amplification were designed using extension times allowing of an amplicon ~750 bp in size, the expected size of an allele containing our targeted large deletion, but not for the WT allele amplicon of 2455 bp (Figure 1A). By increasing the extension time, or including a primer within the predicted deletion site, WT alleles were amplified, ruling out false negatives. Genotyping both single and pooled F0-injected embryos demonstrated that large deletions were efficiently generated with simultaneous injection of two CRISPR gRNAs, along with *cas9* mRNA (Figure 1B). 

Using this strategy, we were able to identify F0 adults with germline transmission of *lats2* large deletions. This led to the generation of F1 fish with a *lats2* mutant allele containing an in -frame 1710-bp deletion. This deletion corresponds to a 570-amino-acid deletion in the Lats2 protein. We refer to this mutant allele as *lats2*^mw87^. This was the only germline-transmitting mutant allele recovered. A previously reported *lats2* nonsense mutant allele containing a 16-bp deletion in exon 3 (termed *lats2*^ncv108^) has been described. Fish homozygous for this allele were reported as viable, with no obvious defects [37]. Interestingly, we find that homozygous *lats2*^mw87^ fish generated from heterozygous incrosses appear viable, with no overt phenotypes at five days post-fertilization (dpf); however, by 60 dpf we fail to recover Mendelian ratios of *lats2*^mw87^ homozygous mutants, with a significant discrepancy between the observed and expected genotype proportions (*p* < 0.0025; Chi-square analysis; Table 1). The few *lats2*^mw87^ homozygous mutants that did survive to adulthood appeared to be largely normal; however, all died prior to reaching 12 mpf, with no identifiable cause of death, and we were unable to breed these animals. One explanation for the potential differences in viability between these *lats2* alleles could be the recently described genetic compensation observed in several cases of mutant alleles containing indels and a premature termination codon [38,39]. In these cases, it appears that the nonsense-mediated decay caused by premature termination codons is able to initiate compensatory increases in gene expression of known orthologues, and thus mask potential phenotypes. As the *lats2^ncv108^* is a nonsense frameshift mutation, it may trigger this compensatory mechanism, with *lats1* being a prime candidate. Our in-frame deletion would not be predicted to trigger this effect. Alternatively, our large deletion could result in a truncated Lats2 protein that functions in a dominant negative fashion and thus caused the more severe decrease in viability we observed. Although they are not the focus of this study, the discrepancies between these two mutant alleles could provide interesting insight into Lats2 function and warrant further investigation.

As F0 fish, mosaic for deletion of *lats2*, grew to adulthood, we found that as early as three months post-fertilization (mpf), fish developed large tumors (Figure 1C–H’). These tumors varied in location across both the head and the torso of the fish. The tumors observed also varied in the degree of pigmentation they contained; some appeared primarily opaque with scattered pigmentation, and others were more darkly pigmented. By 6 mpf, ~14% (13/96) of surviving *lats2* CRISPR-injected fish developed visible tumors. At 12 mpf the percentage of tumor incidence increased to ~24% (23/96) (Figure 1I). Notably, we identified tumors only by gross observation of free swimming fish in tanks. Therefore, the tumor incidence could likely be higher than the numbers reported here. Similarly, the initial onset of these tumors was likely to occur earlier than our first detection of obvious tumors. Indeed, tumors showed variability in size, making it likely that we were unable to identify smaller, internal tumors. Most often we observed one prominent tumor on an affected fish. However, we did identify some individuals where two separate tumors were visible. Of note, we did not observe any tumor formation in the few *lats2*^mw87^ homozygous mutants that survived to adulthood.

**Figure 2:**
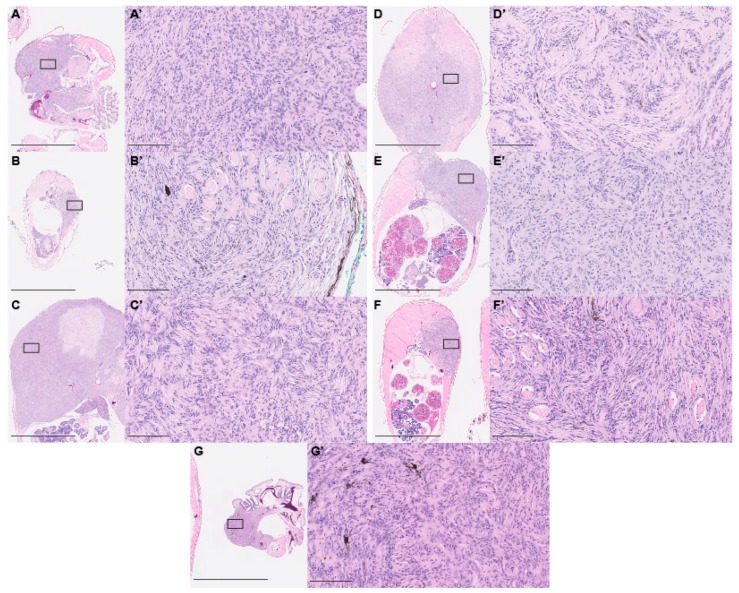
*lats2* CRISPR-injected fish develop tumors resembling PNSTs. (**A**–**G**) Low power image of tumors revealed anatomical position and size. Scale = 2.5 mm F and G represent two distinct tumors from the same fish. (**A’**–**F’**) Higher-power images of the sections in (**A**–**F**) revealing cellular morphology. Scale = 100 μm. Boxes in **A**–**G** denote the tumor area shown at higher magnification in **A’**–**G’**.

We isolated six fish containing tumors of varying sizes and locations for detailed histology. We utilized hematoxylin- and eosin-stained paraffin sections for this analysis. Histology revealed that every tumor exhibited very similar spindle cell morphology, and extensively infiltrated both muscle and bone. Spindled cells were observed wrapping around muscle cell fibers in each fish analyzed (Figure 2A–F’). Histological analysis suggested that these were PNSTs. More specifically, the whorl pattern seen in the tumors, termed a “Schwannian whorl,” is a distinct characteristic of cellular schwannomas [40]. Tumors also contained varied numbers of pigment-bearing cells, scattered throughout the tumor area. While rare, cases of PNSTs with pigmented cells have been reported [41]. Given that both melanocytes and Schwann cells arise from Sox10-positive neural crest lineage, the tumors could originate from an early neural crest lineage. As Hippo signaling has been implicated in cancers of multiple type and origin, we wondered whether other tissues might also be affected. To assess this, we dissected fish containing visible tumors and assessed all tissues for tumor nodules. We also performed histological analysis and were unable to identify any tumors other than the described PNSTs. As we did not perform this detailed analysis on every *lats2* CRISPR-injected fish, it is possible that we missed rare tumors from other origins or tissues. However, the fact that all identified tumors were PNSTs suggests that Hippo pathway signaling is of particular importance in PNST biology.

**Figure 3:**
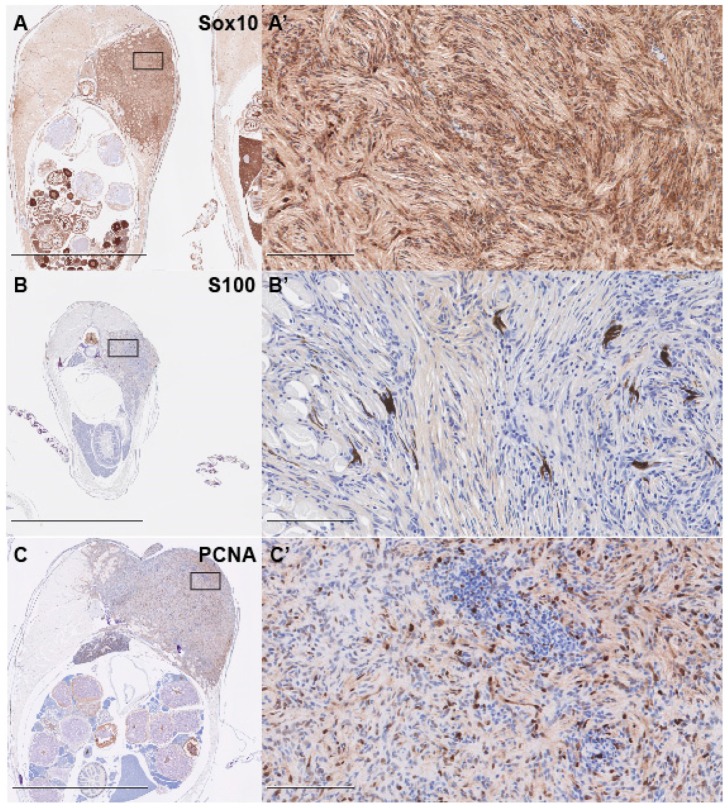
*lats2* CRISPR-injected fish tumors bear markers supporting PNST diagnosis. (**A**,**A’**) Representative images of Sox10 immunohistochemical staining in *lats2* CRISPR-injected fish. (**B**,**B’**) Representative images of S100 immunohistochemical staining in *lats2* CRISPR-injected fish. (**C**,**C’**) Representative images of PCNA immunohistochemical staining in *lats2* CRISPR-injected fish Scale = 2.5 mm for **A**–**C**, and 100 μm for **A’**–**C’**. Boxes in **A**–**C** denote the tumor area shown at higher magnification in **A’**–**C’**.

To confirm the diagnosis of PNST, we performed immunohistochemical (IHC) staining with two known markers of PNSTs, S100 and Sox10 [40,42,43]. We found that tumors were largely Sox10-positive, while diffusely and faintly positive for S100, supporting the diagnosis of PNST (Figure 3A–B’). IHC staining for proliferative cell nuclear antigen (PCNA) revealed that the tumors were highly proliferative, with large numbers of cells throughout the tumors staining positive (Figure 3C,C’).

**Figure 4:**
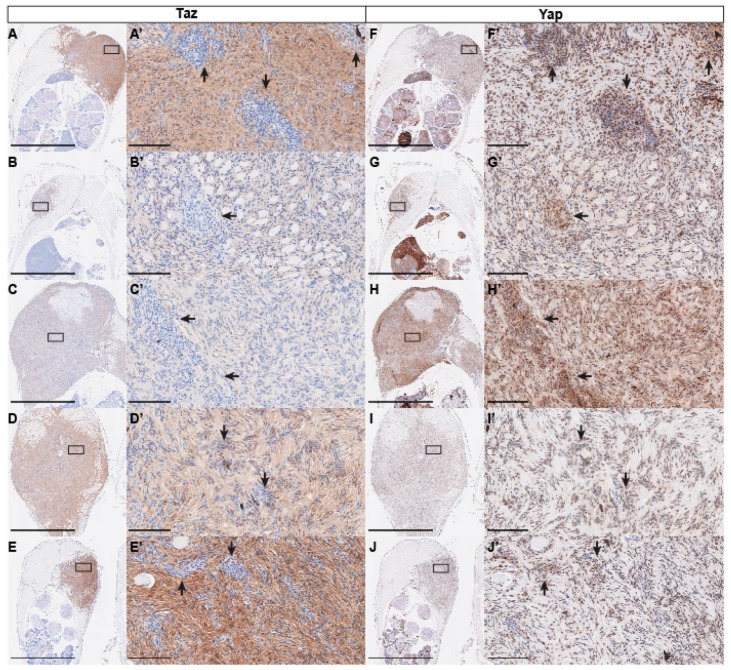
Yap/Taz protein expression in *lats2* CRISPR-injected fish tumors. (**A**–**E**, **A’**–**E’**) Representative images of Taz immunohistochemical staining in *lats2* CRISPR-injected fish. (**F**–**J**, **F’**–**J’**) Representative images of Yap immunohistochemical staining in *lats2* CRISPR-injected fish. Arrows denote regions of high cell density in **A’**–**J’**. Scale = 2.5 mm for **A**–**J**, and 100 μm for **A’**–**J’**. Boxes in **A**–**J** denote the tumor area shown at higher magnification in **A’**–**J’**.

**Figure 5:**
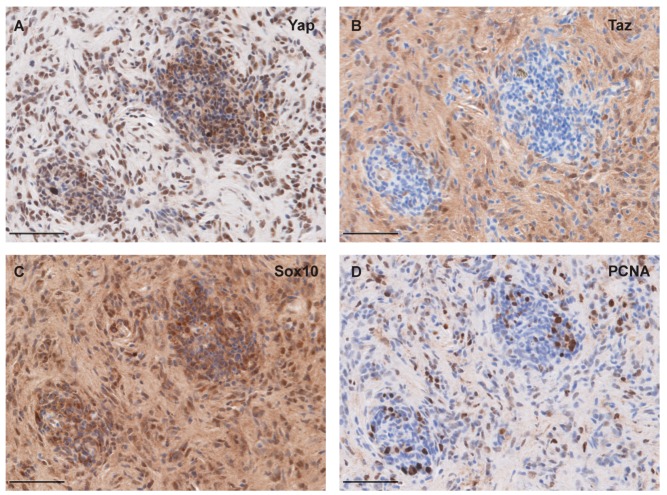
Yap/Taz, Sox10 and PCNA protein expression in areas of high cell density. (**A**) Representative image of Yap immunohistochemical staining in regions of high cell density with peripheral nerve sheath tumor. (**B**) Adjacent section to A with Taz immunohistochemical staining. (**C**) Adjacent section to A with Sox10 immunohistochemical staining. (**D**) Adjacent section to A with PCNA immunohistochemical staining. Scale = 50 μm.

Yap and Taz are the main effectors of the Hippo pathway, and the targets of Lats1/2 phosphorylation. As phosphorylation of Yap and Taz by Lats1/2 leads to their cytoplasmic sequestration and eventual ubiquitin-mediated degradation, we also assessed Yap and Taz protein by IHC. To assay the Taz protein, we used a Yap/Taz antibody that we and others have previously shown to specifically recognize Taz in zebrafish [44,45,46]. Staining with this antibody revealed that all tumors analyzed were positive for Taz; however, we did see variability in the intensity of staining from sample to sample (Figure 4A–E). Closer inspection shows that, while most tumor cells show Taz staining, Taz was often primarily cytoplasmic and diffuse, with scattered cells displaying nuclear Taz. We also observed that areas of high cell density appeared to correlate to low or absent Taz staining (Figure 4A’–E’). Similar to Taz, staining for Yap revealed high levels of staining in all tumor samples (Figure 4F–J). In contrast to Taz staining, we found that Yap staining appeared more consistently nuclear. Most interestingly, the same areas of high cellularity that show low or absent Taz were consistently Yap-positive, and in some cases appeared to be enriched (Figure 4F’–J’). The variation in staining pattern between these two antibodies suggests that there may be differences between Yap and Taz and the forms in which they are found in PNST (i.e., nuclear vs. cytoplasmic, phosphorylated vs. unphosphorylated, etc.), and perhaps define different cell states within the tumor. To further assess these regions of high cell density, we assessed Sox10 and PCNA expression on adjacent sections. We found that these regions did not appear to be significantly different from the surrounding tissue in Sox10 or PCNA staining, suggesting that their identity and proliferative capacity are not greatly altered (Figure 5A–D). While Yap and Taz were found to have many redundant functions, compound genetic studies indicate that Taz may play a more prominent role in Schwann cell development and myelination [16,17,18]. This suggests that in PNST tumorigenesis, Yap and Taz could share many functions, but also display divergent roles. Our results showing the exclusion of Taz staining within regions of high cell density has yet to be described in existing models of PNST, which suggests that, within single PNSTs, separate populations of cells exist with distinct Hippo-Yap/Taz signaling states.

Our study follows the publication of a similar report that found that conditional deletion of *Lats1/2* within the Schwann cell lineage led MPNST formation in mice [29]. The results presented here from zebrafish are largely consistent with the findings in mice, and therefore demonstrate a conserved role for Lats2 signaling in PNSTs across species. However, we note several differences between these two models. In the mouse model, knockout of *Lats1* or *Lats2* alone was insufficient for tumor formation. Zebrafish consistently developed tumors with somatic targeting of *lats2* alone. Importantly, our CRISPR gRNAs are targeted against regions specific to the *lats2* gene, and do not target *lats1* sequences. We also targeted *lats1* with a similar large deletion strategy using separate gRNAs and found that 0 of 33 F0 fish developed tumors (Figure S1). Interestingly, the earliest we identified tumorigenesis in the fish was 3 mpf, with some fish first showing visible tumors at one year of age, and still other fish never developing tumors. Conversely, Wu et al. found that inactivation of Lats1/2 in mice led to rapid tumor formation, with palpable tumors developing as early as three weeks of age and a maximum lifespan of 4–5 months [29]. This is particularly intriguing as genetic editing in our fish model likely occurs within the first 24 h post-fertilization. There are several potential explanations for the varying onset of tumorigenesis between these two models of PNST. The delayed tumorigenesis in the fish may be due to compensation by *lats1* in our model. Alternatively, the mosaic nature of our mutagenesis, including heterozygous and homozygous deletions, may result in fewer potential tumor of origin cells, while the Cre recombinase strategy used in the mouse can efficiently create homozygous mutations throughout the Schwann cell lineage. The temporal difference in tumorigenesis may also suggest that, in the fish model, additional mutations in other tumor suppressors or oncogenes may be necessary. We find this explanation most interesting. If it is true, further study of this model focused on identifying the co-occurring mutations necessary for tumorigenesis could prove beneficial.

The data presented here indicate that somatic deletion of *lats2* in zebrafish can serve as a complementary experimental system to the previously described mouse model for probing the mechanisms of Lats2-Yap/Taz PNST. One advantage of the zebrafish model, in comparison with the murine system, is the reduced amount of breeding required to generate large pedigrees and achieve high experimental sample sizes. Injections into large numbers of embryos allows for easy generation of a high number of tumor-bearing fish on any number of genetic or transgenic backgrounds. Another advantage is the increased number of transgenic fish available for characterization of cell signaling, stress responses, or metabolic state. For instance, injection into fluorescent reporter fish for Notch [47,48], BMP [49,50,51], Wnt [52,53], Hedgehog [54,55], Hippo [44,56,57], or other pathways would allow for sorting of tumor cells by reporter activity and subsequent analysis of the unique transcriptomic or proteomic signatures of those populations. Finally, as an aquatic species, drug screens and chemotherapeutic analysis is more easily accomplished.

In conclusion, we present here a novel model of PNSTs in zebrafish by somatic inactivation of the Hippo kinase Lats2. This methodology shows robust presentation of PNST formation in adult genetically mosaic fish. This work further solidifies the role of Hippo-Yap/Taz signaling, and specifically Lats2 in PNST biology, and offers a new model to study this form of cancer.

## Figures and Tables

**Table 1:** *lats2*^WT/mw87^ in-cross survival and Chi-square analysis.

	*lats2* ^WT/WT^	*lats2* ^WT/mw87^	*lats2* ^mw87/mw87^
Expected	14.25 (25%)	28.50 (50%)	14.25 (25%)
Observed	19 (33.33%)	35 (61.40%)	3 (5.26%)
Chi-square	11.95		
Degrees Freedom	2		
*p* value (two tailed)	0.0025		

## Supplementary Materials

The following are available online at https://www.mdpi.com/2073-4409/8/9/972/s1, Figure S1.
