# Supplementary material for: Somatic Mutations of lats2 Cause Peripheral Nerve Sheath Tumors in Zebrafish
Source: Cells. 2019 Aug 25;8(9):972. doi: 10.3390/cells8090972 (PMC6770745; doi:10.3390/cells8090972)
Supplement: Supplementary file 1 [file cells-08-00972-s001.pdf]

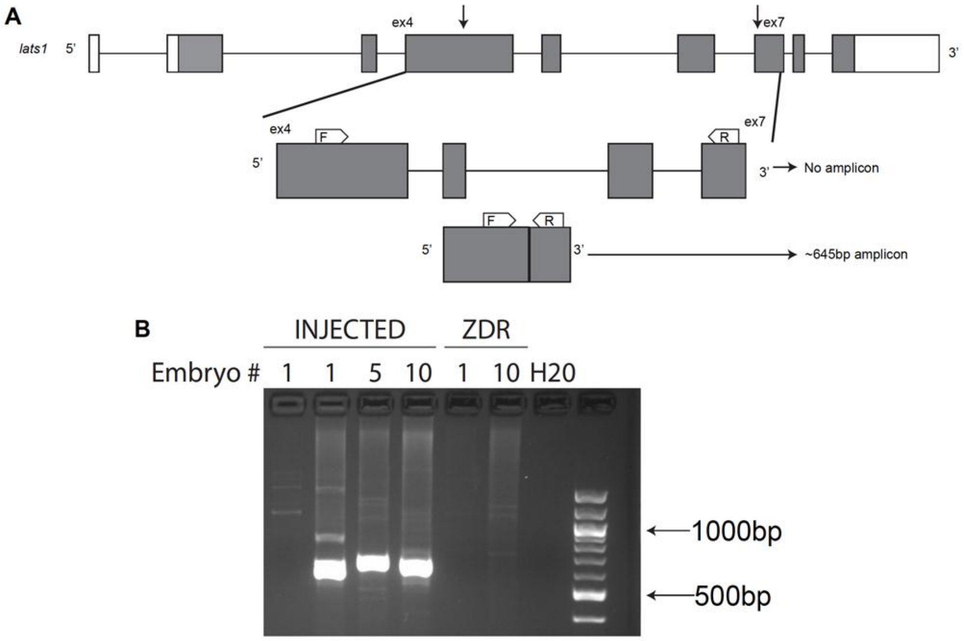

Supplemental Figure 1. Injection of two *lats1* gRNAs leads to efficient *lats1* large deletions (**A**) Schematic depicting the zebrafish *lats1* gene and the PCR based genotyping assay used to identify and differentiate between successful large deletion alleles and WT alleles. Arrows indicate the target sites of the gRNAs used. F refers to forward primer; R refers to reverse primer. (**B**) Agarose gel separation of PCR amplicons generated based on genotyping assay depicted in **A**. Amplicons of the correct size, indicative of a large genomic deletion, are present in *lats1* CRISPR injected embryos, but absent in un-injected negative control ZDR fish strain. Numbers above lanes represent the number of embryos used as the template DNA for each PCR sample. Last lane contains 100bp ladder (NEB).
